# Multiple components of the nuclear pore complex interact with the amino-terminus of MX2 to facilitate HIV-1 restriction

**DOI:** 10.1371/journal.ppat.1007408

**Published:** 2018-11-29

**Authors:** Matthew D. J. Dicks, Gilberto Betancor, Jose M. Jimenez-Guardeño, Lucie Pessel-Vivares, Luis Apolonia, Caroline Goujon, Michael H. Malim

**Affiliations:** Department of Infectious Diseases, School of Immunology & Microbial Sciences, King’s College London, London, United Kingdom; Fred Hutchinson Cancer Research Center, UNITED STATES

## Abstract

Human myxovirus resistance 2 (MX2/MXB) is an interferon-induced post-entry inhibitor of human immunodeficiency virus type-1 (HIV-1) infection. While the precise mechanism of viral inhibition remains unclear, MX2 is localized to the nuclear envelope, and blocks the nuclear import of viral cDNAs. The amino-terminus of MX2 (N-MX2) is essential for anti-viral function, and mutation of a triple arginine motif at residues 11 to 13 abrogates anti-HIV-1 activity. In this study, we sought to investigate the role of N-MX2 in anti-viral activity by identifying functionally relevant host-encoded interaction partners through yeast-two-hybrid screening. Remarkably, five out of seven primary candidate interactors were nucleoporins or nucleoporin-like proteins, though none of these candidates were identified when screening with a mutant RRR11-13A N-MX2 fragment. Interactions were confirmed by co-immunoprecipitation, and RNA silencing experiments in cell lines and primary CD4^+^ T cells demonstrated that multiple components of the nuclear pore complex and nuclear import machinery can impact MX2 anti-viral activity. In particular, the phenylalanine-glycine (FG) repeat containing cytoplasmic filament nucleoporin NUP214, and transport receptor transportin-1 (TNPO1) were consistently required for full MX2, and interferon-mediated, anti-viral function. Both proteins were shown to interact with the triple arginine motif, and confocal fluorescence microscopy revealed that their simultaneous depletion resulted in diminished MX2 accumulation at the nuclear envelope. We therefore propose a model whereby multiple components of the nuclear import machinery and nuclear pore complex help position MX2 at the nuclear envelope to promote MX2-mediated restriction of HIV-1.

## Author summary

The movement of large molecules into the cell nucleus is regulated at specific sites within the nuclear envelope termed nuclear pores. To infect cells productively, human immunodeficiency virus type-1 (HIV-1) must traverse the nuclear envelope to enable integration of the viral DNA into the genomic DNA of host cells. We, and others, have previously identified a cell-encoded protein, human myxovirus resistance 2 (MX2), which is expressed upon initiation of an innate immune response and prevents accumulation of HIV-1 DNA within the nucleus, thus imposing a block to infection. Here, we reveal that components of the nuclear pore complex, and nuclear import machinery, are required for MX2-dependent inhibition of HIV-1 infection. We show that MX2, which is localized at the cytoplasmic face of the nuclear envelope, interacts with multiple protein components of the nuclear pore complex, as well as transport receptor transportin-1, via a functionally required triple arginine motif at its amino-terminus. We speculate that these interactions facilitate MX2-mediated inhibition of HIV-1 nuclear import by situating the protein at the nuclear envelope.

## Introduction

Human myxovirus resistance 2 (MX2/MXB) is an interferon-stimulated gene (ISG), and a key contributor to the type-1 interferon-induced post-entry inhibition of human immunodeficiency virus type-1 (HIV-1) infection [1–3]. The MX2-mediated block to HIV-1 infection occurs after reverse transcription, but prior to the nuclear import of pre-integration viral nucleoprotein replication complexes [1, 2]. The precise mechanism of inhibition remains unclear, although the HIV-1 Capsid protein (CA) is believed to determine viral specificity since point mutations in CA can allow escape from MX2-mediated inhibition [1–4]. Furthermore, MX2 has been shown to interact with synthetic Capsid-Nucleocapsid nanotubes *in vitro*. However, the relationship between CA binding and viral suppression is not straightforward, since mutations in CA that permit escape from viral inhibition do not prevent CANC nanotube interactions with MX2 [5–7].

Human MX2 is a large dynamin-like GTPase, and is most closely related to human MX1/MXA. MX1 has long been established as a potent interferon-induced restriction factor for a range of DNA and RNA viruses including influenza A virus (IAV), though it does not inhibit retroviruses such as HIV-1 [8, 9]. Indeed, despite sharing 63% amino acid sequence identity and a similar structure and domain architecture [5], the viral specificities and mechanisms of action appear to differ considerably between MX1 and MX2. While GTPase activity is essential for the anti-viral function of MX1 against IAV [10, 11], inactivating mutations in conserved residues within the GTPase-domain of MX2 do not abrogate anti-viral activity against HIV-1 [1, 2]. Both MX1 and MX2 can oligomerize, forming a variety of multimeric species from dimers and trimers to high-order oligomers [8, 12, 13], and recombinant MBP-tagged MX2 fusion proteins can form large helical assemblies *in vitro* [14]. In a recent structure-function study by Gao *et al*, higher-order oligomerization of MX1 was found to be essential for anti-viral activity against IAV [15]. However, while monomeric MX2 mutants are not anti-viral, higher-order oligomerization appears to be dispensable for MX2-mediated inhibition of HIV-1 [5, 13, 16].

MX2 possesses an extended N-terminal domain that distinguishes it from MX1, and exists as two isoforms owing to the presence of an internal initiation methionine codon at position 26. However, only the longer 78 kDa isoform displays anti-HIV-1 activity and is associated with the nuclear envelope (NE); the shorter 76 kDa isoform is cytoplasmic (as is human MX1), and is not anti-viral. Indeed we, and others, have previously shown that the N-terminal 91 amino acids of full-length MX2 are required for anti-viral activity [17, 18], and remarkably can confer anti-HIV-1 activity on heterologous scaffolds including MX1, the murine leukemia virus (MLV) restriction factor Fv1, and oligomerization-competent leucine-zippers [19]. Previously, we identified a triple-arginine motif at positions 11 to 13 as essential for the anti-HIV-1 activity of MX2 [19]. Mutation of these three residues, either to alanine (RRR11-13A), or lysine (RRR11-13K), almost entirely abrogate anti-viral function, without observably disturbing NE localization [19]. This motif has also been implicated in binding to HIV-1 CA (20), though its precise role in virus suppression has remained unclear.

To address the role of the N-terminal domain in MX2 function, we therefore sought to identify biologically relevant host-encoded binding partners using yeast two-hybrid (Y-2-H) screening. Strikingly, most of the candidate interactors with the wild-type N-terminal sequence were nucleoporins (NUPs) or nucleoporin-like proteins. We then determined the functional importance of these interactions using a combination of biochemical, genetic and virological experiments: specifically, we confirmed these interactions through co-immunoprecipitation assays, and used RNA silencing-mediated depletion to demonstrate that multiple components of the nuclear pore complex (NPC) and the nuclear import machinery promote MX2-mediated inhibition of HIV-1 infection.

## Results

### Multiple components of the nuclear pore complex interact with MX2 through its N-terminal RRR^11-13^ motif

**Fig 1:**
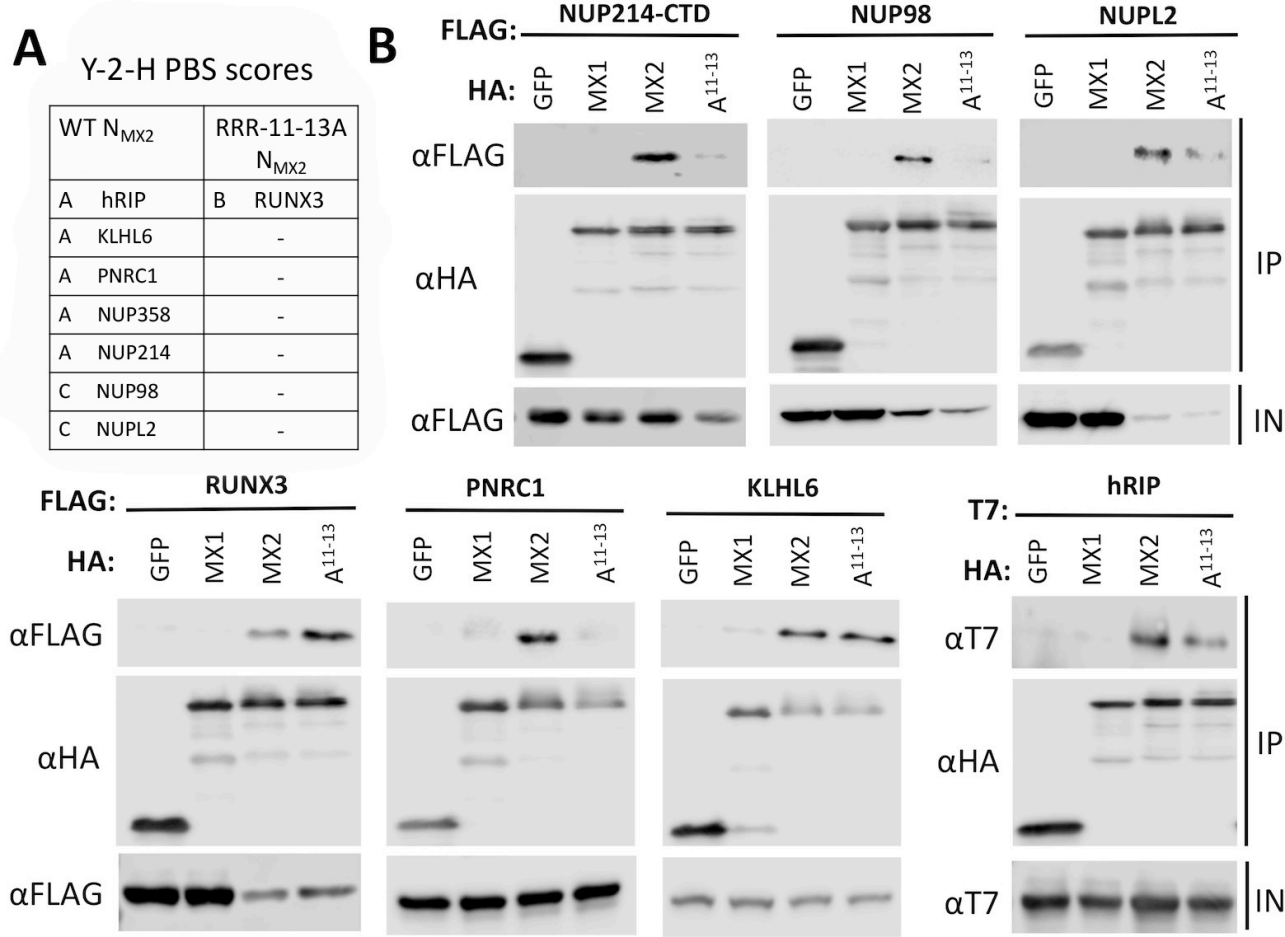
Multiple nucleoporins interact with the N-terminal domain of MX2 (N-MX2). **(A)** A yeast two-hybrid (Y-2-H) screen was performed using a human leukocyte cDNA library to identify interacting proteins with wild-type or mutant RRR11-13A N-MX2. Interacting partners were assigned a predicted biological score from A-F to assess the confidence of an interaction being specific (with A indicating very high confidence, and F indicating experimentally determined artifacts). **(B)** Co-immunoprecipitation of candidate interacting proteins with MX2. 293T cells were co-transfected with HA-tagged wild-type or mutant RRR11-13A MX2 and FLAG-tagged candidate interactors from the Y-2-H screen. Cells were lysed and HA-tagged protein immunoprecipitated with anti-HA antibody. Co-transfection of FLAG-tagged candidates with HA-tagged MX1 or GFP served as negative controls. Immunoblots of immunoprecipitated proteins (IP) were probed with anti-FLAG and anti-HA antibodies. As a control for protein expression, samples of lysate prior to immunoprecipitation (IN) were probed with anti-FLAG antibody. All experiments were performed at least three times.

A yeast two-hybrid screen was performed to identify candidate interaction partners with the MX2 N-terminal domain (N-MX2). The N-terminal 91 amino acids of wild-type MX2, or mutant RRR11-13A MX2, were employed as bait fragments to screen against prey fragments from a human leukocyte cDNA library. Candidate interactors were assigned a Predicted Biological Score (PBS) (from A to F; A representing a very high confidence in the interaction, F representing experimentally proven artifacts as previously described [21]). Candidate interactors with PBS scores of A, B or C with wild-type N-MX2 or mutant RRR11-13A N-MX2 are shown in Fig 1A, with the complete lists of all identified genes displayed in S1 Table. For wild-type N-MX2, five out of the seven candidate interactors are either FG-nucleoporins (nucleoporins containing phenylalanine-glycine repeat sequences; NUP358, NUP214 and NUP98) or nucleoporin-like proteins containing FG-repeats (NUPL2/hCG1 and human Rev-interacting protein/hRIP). However, none of these proteins were recovered using the mutant RRR11-13A N-MX2 as bait.

To validate these interactions, co-immunoprecipitation analyses were performed by co-expression of hemagglutinin (HA)-tagged wild-type MX2 or RRR11-13 mutant MX2 and FLAG-tagged candidate interactors in 293T cells, HA-specific immunoprecipitation, and immunoblot detection of associated FLAG-tagged proteins (Fig 1B). These data largely corroborated the interactions from the Y-2-H screen. NUP98, a 35 kDa C-terminal domain of NUP214 (the predicted site of interaction from the Y-2-H screen) and PNRC1 all exhibited a clear interaction with wild-type MX2, but almost no interaction with RRR-11-13A or the negative controls MX1 and green fluorescent protein (GFP) (S2 Table). hRIP exhibited a stronger interaction with MX2 than RRR-11-13A (and virtually no interaction with the negative controls) while KLHL6 and NUPL2 exhibited a comparable interaction with MX2 and RRR-11-13A. RUNX3, a predicted interactor of RRR-11-13A but not wild-type MX2, did indeed exhibit a stronger interaction with RRR-11-13A than wild-type MX2. Multiple experimentally varied co-immunoprecipitation studies with the regions of NUP358 predicted to interact with MX2 were unsuccessful.

### Nuclear pore complex components and nuclear import factors are required for MX2-mediated inhibition of HIV-1

NUP358, NUP214, NUP98 and NUPL2 have been described as cytoplasmic filament nucleoporins [22] and have all been previously implicated in HIV-1 infection [23–29]. These proteins are all components of the NPC and are localized, at least partially, at the cytoplasmic face of the NE. They all possess multiple FG-repeats, which have been proposed to act as sequential docking sites for nuclear transport receptors as they traverse the NPC [30], facilitating nuclear import/export of associated cargo (reviewed in [31]). MX2 imposes a barrier to HIV-1 infection that follows reverse transcription but precedes nuclear import of the viral cDNA [1]. These nucleoporins and hRIP (which also resides on the NE cytoplasmic face) are, therefore, conceptually well placed to contribute to regulatory processes that influence HIV-1 nuclear import.

**Fig 2:**
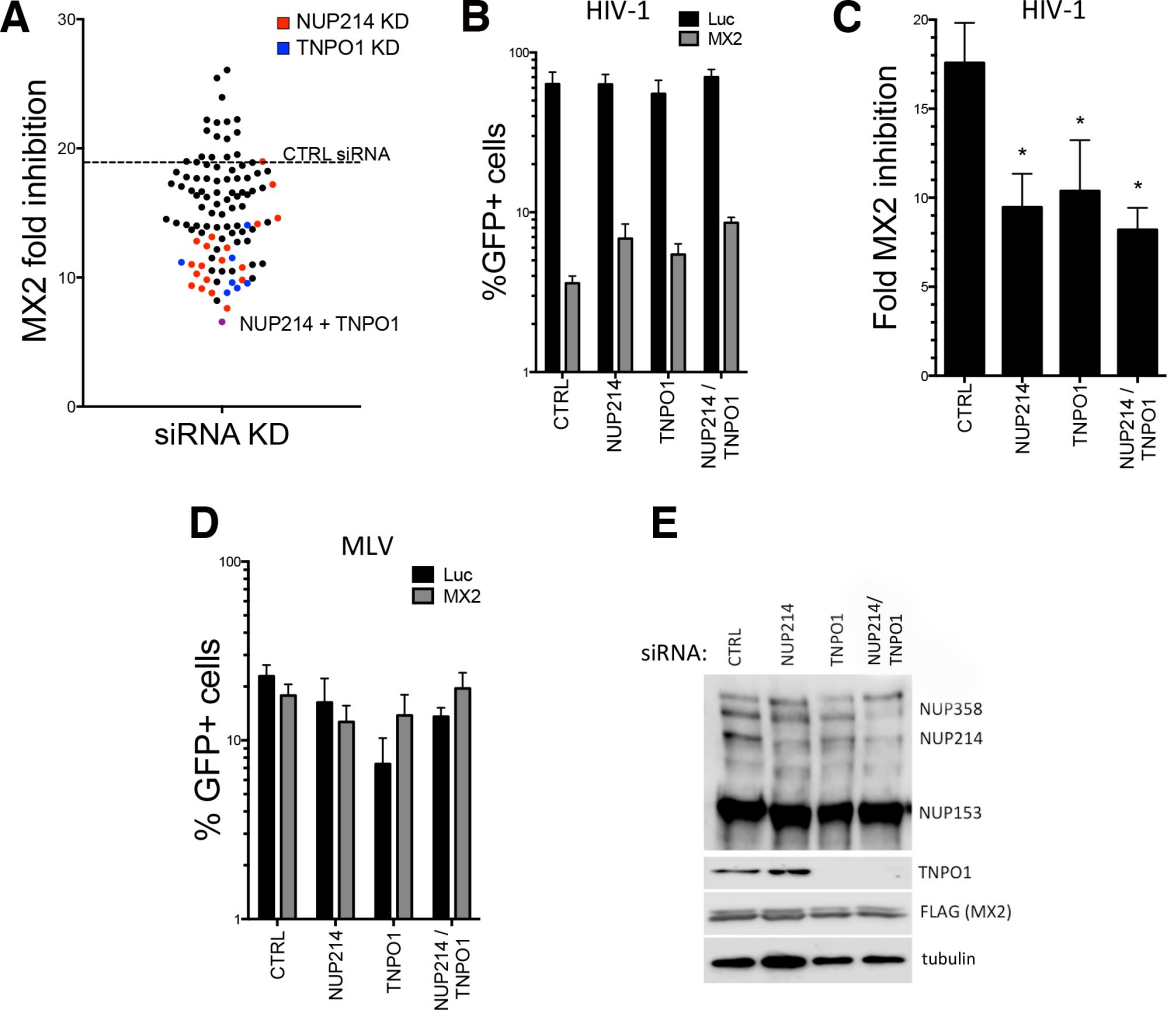
NUP214 and TNPO1 are required for full anti-viral activity of MX2 in U87-MG cells. U87-MG CD4^+^ CXCR4^+^ cells were transduced with EasiLV vectors expressing FLAG-tagged MX2 or Luciferase (control). After 48 h, transduced cells were transfected twice, 24 h apart, with 20 nM of specific siRNAs (a non-targeting siRNA was included as a control, CTRL). Expression of MX2 or Luciferase was then induced by treatment of cells with doxycycline (0.5 μg/ml) for ~72 h prior to challenge with a HIV-1 based lentiviral vector expressing GFP (HIV-1/GFP). Transduction efficiency was assessed 48 h post challenge by flow-cytometry. **(A)** NUP358, NUP214, NUP98, hRIP, PNRC1, KLHL6, NUP62, NUP153, TNPO1 or TNPO3 were depleted independently or in pairs and MX2 anti-viral activity was analyzed. A dotted line indicates the fold inhibition of HIV-1 obtained in cells treated with CTRL siRNA. Conditions where NUP214 or TNPO1 where depleted are highlighted in red and blue, respectively, while depletion of both is indicated in purple. **(B-C)** Effect of siRNA-mediated depletion of NUP214 and/or TNPO1 on the anti-HIV-1 activity of MX2. In C, the same data as in B are represented as MX2-mediated fold inhibition by dividing %GFP+ luciferase expressing cells by %GFP+ MX2 expressing cells (n = 3; mean ± standard deviation (SD); *p-value < 0.05; paired t-test to CTRL siRNA). **(D)** The effect of NUP214 and/or TNPO1 depletion on MLV infectivity in the presence of MX2 was studied by challenging siRNA-transfected cells with an MLV based vector expressing GFP. **(E)** Immunoblot analysis of MX2 expressing cells from the experiment described in B-D, indicating levels of FLAG-tagged MX2 and endogenous NUP214 (detected using mab414) and TNPO1 after siRNA treatment. Tubulin is included as a loading control.

We investigated whether these candidates, as well as selected other nuclear import factors and NPC components, are functionally linked with MX2 anti-viral activity. A focused siRNA “screen” incorporating other nucleoporins previously implicated in HIV-1 infectivity such as NUP153 [32, 33], NUP62 [34, 35] and transport receptor transportin-3 (TNPO3, also referred to as TRN-SR2) [36, 37], as well as the related transportin-1 (TNPO1, also called karyopherin-β2/KPNB2), was therefore performed. FLAG-tagged human MX2 and Photinus luciferase (negative control) constructs were overexpressed in U87-MG CD4^+^ CXCR4^+^ cells using the doxycycline-inducible EasiLV lentiviral vector system [1]. Prior to induction of expression, transduced cells were treated with experimental or control siRNAs. Following doxycycline treatment, cells were challenged with an HIV-1 based lentiviral vector (HIV-1/GFP) and transduction efficiency assessed after 48 h by flow cytometry (S1 Fig, Fig 2A). Overexpression of human MX2 exhibited an ~18-fold inhibition of HIV-1/GFP infection compared to the luciferase control after either treatment with a non-targeting control siRNA pool or no siRNA treatment, consistent with previous observations [1, 13, 17, 19].

Depletion of a number of nucleoporins (S2 Fig) increased HIV-1 infection in MX2 expressing cells, indicating partial relief from MX2-mediated inhibition (we were unable to deplete NUPL2 using the siRNA pool tested). NUP214 depletion led to the most consistent and robust diminution in MX2-mediated inhibition, with simultaneous depletion of both NUP214 and TNPO1 exhibiting the most pronounced phenotype: ~6-fold inhibition of HIV-1 by MX2, corresponding to a ~3-fold reduction in MX2-mediated inhibition compared to the non-targeting siRNA control. This observation with NUP214/TNPO1 knock-down was then confirmed in a series of more targeted experiments, with the double depletion consistently reducing the MX2 anti-viral effect by ~2 to 3-fold (Fig 2B and 2C). Critically, MLV infection, which is not susceptible to MX2-mediated inhibition [1], was not affected by the depletion of NUP214/TNPO1 (Fig 2D), and neither was the overall expression of MX2 (Fig 2E). Interestingly, siRNA depletion of nucleoporins in general had a minimal general effect on HIV-1 infectivity in control (luciferase expressing) U87-MG cells, despite previous reports that several of the nucleoporins tested here are important for HIV-1 infection in other cell lines [24, 25, 27, 33] (S1 Fig and Fig 2B).

**Fig 3:**
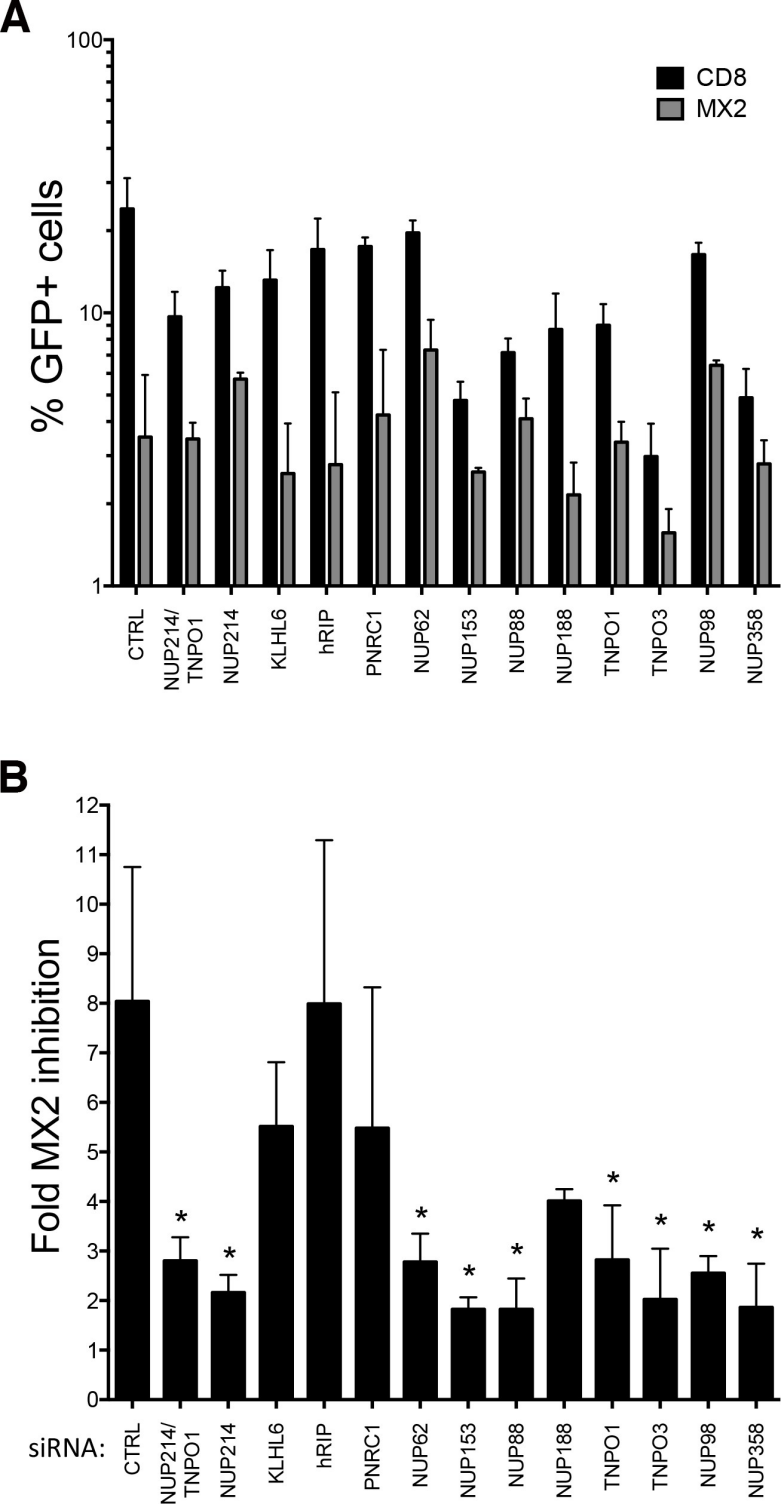
Multiple nucleoporins and transport receptors are required for anti-viral activity of MX2 in HeLa cells. **(A)** HeLa cells were stably transduced with lentiviral vectors constitutively expressing FLAG-tagged MX2 or CD8 (negative control), and transduced cells selected by treatment with 1 μg/ml puromycin for 72 h. After selection, transduced cells were transfected twice, 24 h apart, with 20 nM siRNA targeting individual nucleoporins or transport receptors. A non-targeting siRNA was included as a control, CTRL. After 48 h, cells were challenged with HIV-1/GFP and transduction efficiency assessed by flow cytometry after a further 48 h post challenge. **(B)** the same data as in A are represented as fold MX2-mediated inhibition, calculated as in Fig 2 (n = 3; mean ± SD; *p-value < 0.05; paired t-test to CTRL siRNA).

Due to the observed lack of impact of nucleoporin knockdown on infection of U87-MG cells, we asked whether functional redundancy between nucleoporin-dependent HIV-1 nuclear import pathways in these cells may have occluded the detection of MX2-dependent infection phenotypes. We therefore investigated the nucleoporin requirements for MX2 inhibition in a second cell line, selecting HeLa cells since many previous studies relating to HIV-1 nuclear import have been conducted in this line. Human MX2 or CD8 (negative control) was overexpressed in HeLa cells using a puromycin selectable lentiviral vector system. Puromycin-selected transduced cells were treated with the same targeted siRNA pools used in the previous experiments prior to challenge with HIV-1/GFP, and monitoring of transduction efficiency was performed by flow cytometry (Fig 3).

In notable contrast to the observations in U87-MG cells (S1 Fig and Fig 2), depletion of several nucleoporins reduced HIV-1 infectivity in control (CD8 expressing) cells (Fig 3A). In agreement with previous observations in this cell line, depletion of NUP358 and NUP153 each reduced HIV-1 infectivity by ~5-fold, and depletion of TNPO3 reduced infectivity by ~8-fold, relative to treatment with a non-targeting siRNA). In cells treated with the non-targeting siRNA, MX2 exhibited an ~8-fold inhibition of HIV-1 infection (Fig 3B). Specific siRNA depletion of many of the nucleoporins (confirmed by immunoblotting, S3 Fig) significantly reduced the magnitude of MX2 inhibition (Fig 3B); in particular, silencing of NUP214, NUP62, NUP153, NUP88, NUP358, NUP98 and transportins TNPO1 and TNPO3 each reduced the magnitude of MX2 inhibition of HIV-1 infection to below 3-fold. However, it is important to recognize two caveats with this experiment: 1) the narrowing of the MX2 inhibitory phenotype in HeLa cells silenced for NUP358, NUP153 or TNPO3 is largely driven by reduced infection of control cells rather than the restoration of infection in cells harboring MX2 (i.e., in contrast to what we describe in U87-MG cells, S1 Fig and Fig 2), and 2) the silencing of certain nucleoporins can result in the reduced expression of nucleoporins additional to the intended target (S3 Fig), thus making it impractical to ascribe activity to single proteins in some cases. Notably, knock-down of NUP214, NUP62 or NUP98 had minimal effects (<2-fold) on HIV-1 infection in control cells, indicating that each of these nucleoporins promote MX2 anti-viral function. Depletion of some of the other candidates from the Y-2-H screen such as hRIP, had no effect on MX2-mediated inhibition (Fig 3).

### MX2 interacts with TNPO1, in part via the N-terminal RRR^11-13^ motif

**Fig 4:**
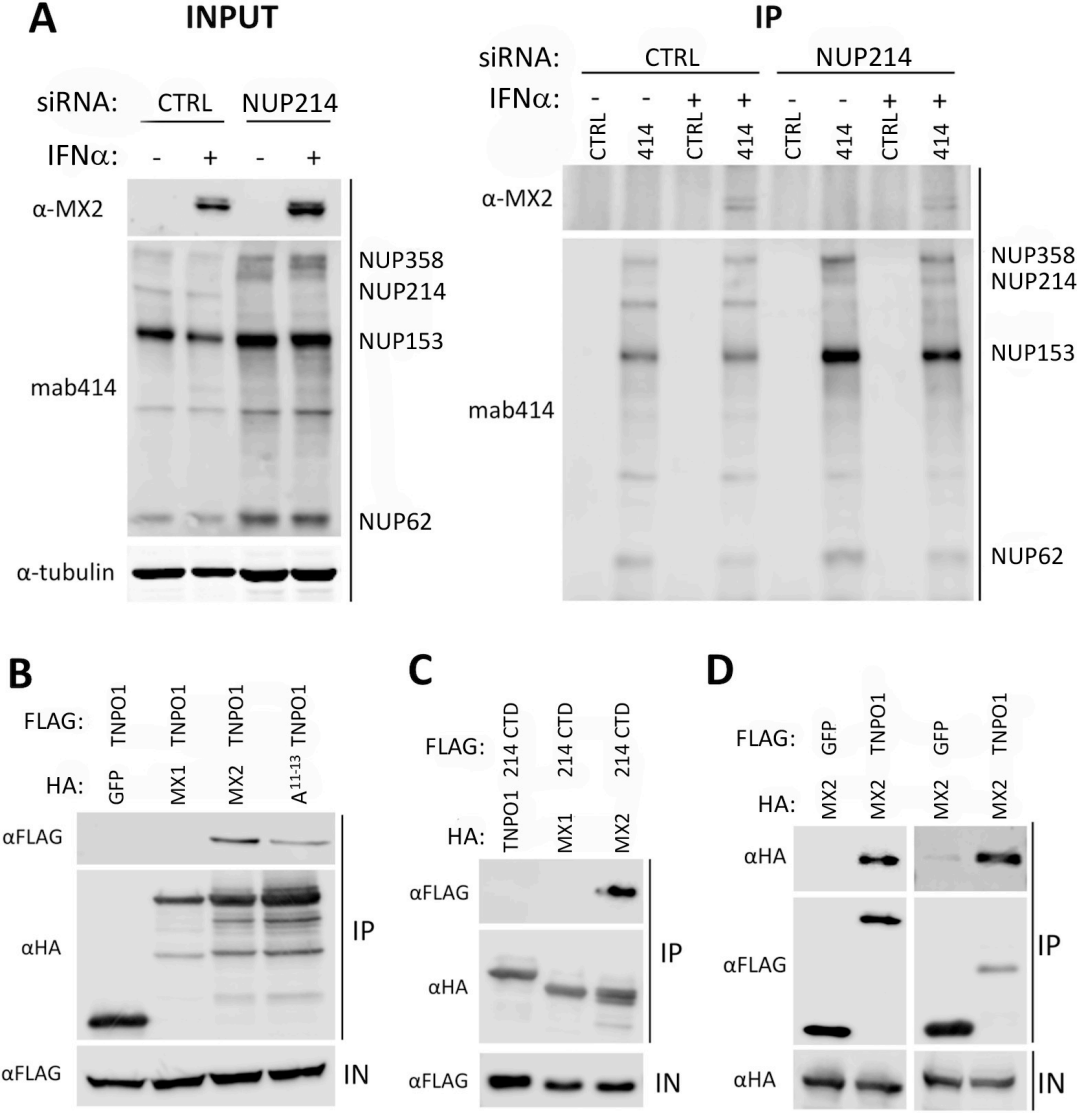
MX2 interacts with TNPO1 via RRR11-13, and endogenous FG-nucleoporins. **(A)** Interaction of MX2 with endogenous FG-nucleoporins. U87-MG CD4^+^ CXCR4^+^ cells were treated with siRNA targeting NUP214 or a non-targeting siRNA (CTRL) as described in Fig 2 and incubated for ~48 h prior to culture with or without IFNα (500 U/ml) for a further 24 h. Treated cells were lysed, and mouse monoclonal mab414 used to extract FG-nucleoporins NUP358, NUP214, NUP153 and NUP62. An anti-GFP mouse monoclonal was included as a control (CTRL). Immunoblots were performed on immunoprecipitated material (IP) to detect the presence of associated MX2, and on samples of lysate prior to precipitation (INPUT). **(B)** TNPO1 interacts with MX2 via RRR11-13. 293T cells were co-transfected with HA-tagged wild-type MX2 or mutant RRR11-13A MX2 and FLAG-tagged TNPO1. Cells were lysed and HA-tagged protein immunoprecipitated with anti-HA antibody. HA-tagged MX1 or GFP were included as negative controls. Immunoblots of immunoprecipitated protein (IP) were probed with anti-FLAG and anti-HA antibodies. As a control for protein expression, samples of lysate prior to immunoprecipitation (IN) were probed with anti-FLAG antibody. **(C)** TNPO1 interacts with MX2 but not the CTD of NUP214. A similar experiment was performed in 293T cells as described in B, except that a FLAG-tagged construct encoding the 35 kDa CTD of NUP214 was co-expressed with HA-tagged constructs expressing TNPO1, MX1 or MX2. Immunoprecipitation was performed with an anti-HA antibody **(D)** MX2 interacts with NUP214 CTD and TNPO1 after FLAG immunoprecipitation. In 293T cells, a similar experiment was performed as described in B and C except that HA-tagged MX2 was co-expressed with FLAG-tagged NUP214 CTD, TNPO1 or GFP and immunoprecipitation of cell lysate performed with an anti-FLAG antibody. All experiments were done at least three times.

We next wished to confirm whether endogenous FG-nucleoporins interact with IFN-induced endogenous MX2. U87-MG cells were first treated with either a non-targeting control siRNA, or anti-NUP214 siRNA followed by the addition of IFNα for 24 h to induce MX2 expression. Immunoprecipitation with mab414, a mouse monoclonal antibody which recognizes FG-repeat regions within multiple nucleoporins including NUP358, NUP214, NUP153 and NUP62 [38], was then performed using cell lysates, and associated MX2 was detected by immunoblotting using an MX2-specific antibody (Fig 4A). Immunoblots of the cell lysate (INPUT) material show efficient IFN-mediated induction of MX2, and specific depletion of NUP214 following siRNA treatment. MX2 was detected in IFN-induced samples immunoprecipitated with mab414, but not with the negative control GFP-specific antibody (Fig 4A), demonstrating an interaction with endogenous FG-nucleoporins. Depletion of NUP214 visibly reduced but did not ablate this interaction (although MX2 levels were noticeably higher in NUP214 siRNA treated samples) implying that at least one other nucleoporin recognized by mab414 can also interact with MX2.

Since NUP214 and TNPO1 were required for full MX2-mediated HIV-1 inhibition in both U87-MG cells and HeLa cells, potential interactions between these proteins and MX2 were explored further. A series of co-immunoprecipitation experiments were performed using 293T cells transfected with HA- and FLAG-tagged constructs, HA-specific immunoprecipitation, and immunoblot detection of associated FLAG-tagged proteins (Fig 4B and 4C). TNPO1 interacts with wild-type MX2, but not GFP or MX1 (Fig 4B), whereas the interaction between TNPO1 and the RRR11-13A mutant is diminished, despite a higher level of RRR11-13A present in the immunoprecipitated material, suggesting that arginine residues 11–13 are important for the interaction (Fig 4B, S2 Table). TNPO1 does not interact with the C-terminal domain of NUP214, though the interaction between NUP214 and MX2 is again demonstrated (Fig 4C). Observed interactions between MX2 and both NUP214 and TNPO1 were also demonstrated by similar experiments performed with FLAG-specific immunoprecipitation instead of HA-specific immunoprecipitation (Fig 4D). Additional attempts to explore the possible formation of a ternary NUP214-MX2-TNPO1 complex were unsuccessful, but perhaps this is to be expected since both interactions depend upon the integrity of the triple-arginine motif at positions 11 to 13 and may therefore be mutually exclusive.

### Assessment of endogenous MX2, NUP214 and TNPO1 function during IFN-mediated inhibition of HIV-1

**Fig 5:**
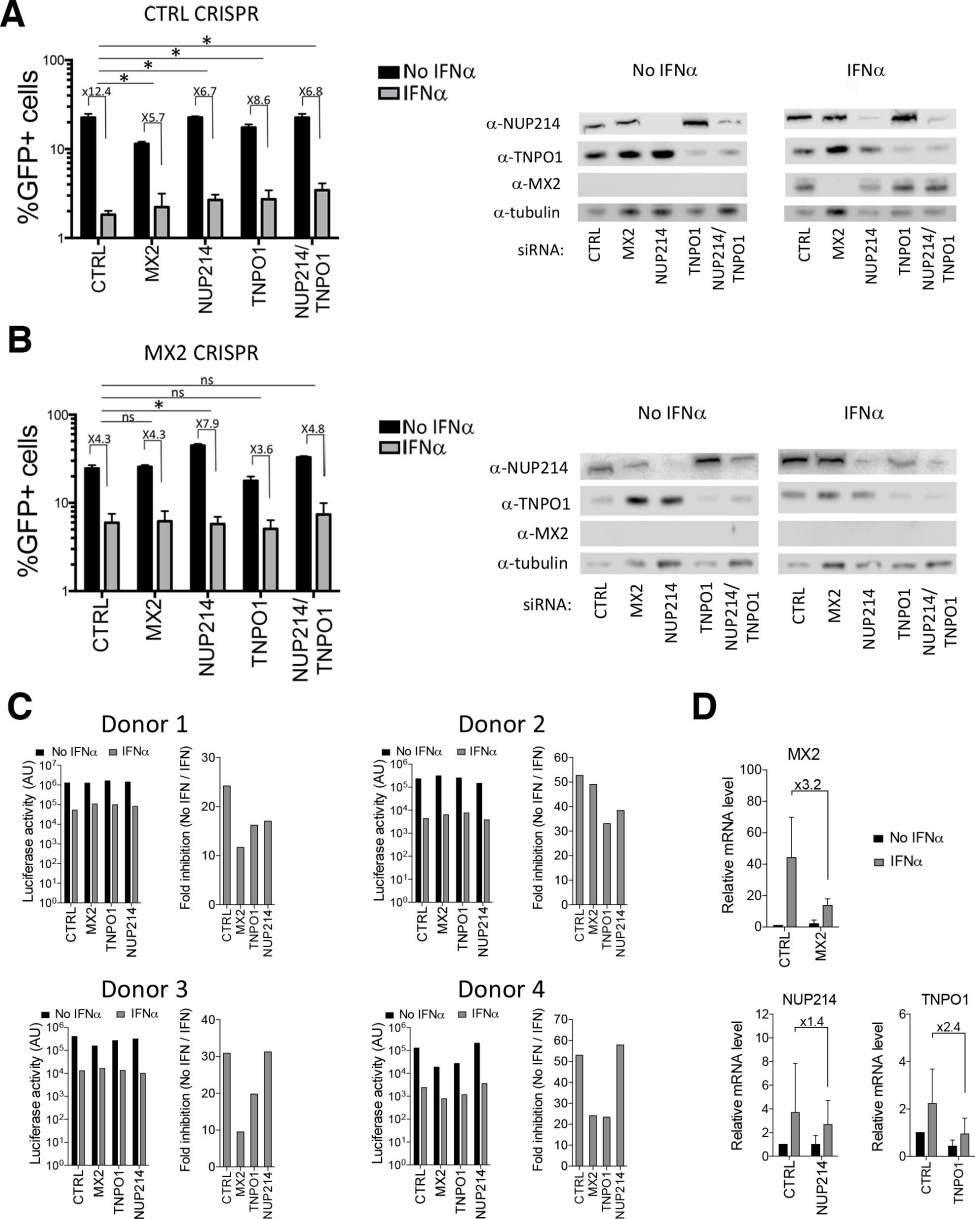
Endogenous MX2 requires NUP214 and TNPO1 for a full anti-viral function. **(A)** U87-MG cells transduced with a CRISPR-Cas9 control guide RNA were transfected twice with a control siRNA (CTRL) or siRNAs targeting MX2, NUP214 and/or TNPO1, and treated or not with 1000 U/ml of IFNα, prior to infection with an HIV-1/GFP lentiviral vector. On the left, the percentage of infected cells calculated by flow cytometry and the fold inhibition of infection due to IFNα treatment are shown (n = 4; mean ± SD; *p-value < 0.05; (ns) non-significant; paired t-test). On the right is an immunoblot from a representative experiment, showing depletion of the indicated proteins. **(B)** Same experiment as in A, but using U87-MG cells where the *MX2* alleles were disrupted using CRISPR-Cas9 genome editing (n = 4; mean ± SD; *p-value < 0.05; (ns) non-significant; paired t-test). **(C)** Primary CD4^+^ T cells were isolated from 4 independent donors, transduced with shRNAs targeting MX2, NUP214, TNPO1 or a control shRNA (CTRL) and treated or not with 3000 U/ml of IFNα. 24 h later, cells were challenged with NL4.3/Nef-IRES-Renilla and luciferase activity determined 48 h later. The mean of three technical replicates are shown for each donor on the left, and the fold inhibition of infection (no IFN/IFN) on the right. **(D)** Efficiency of MX2, NUP214 and TNPO1 depletion in primary CD4^+^ T cells following shRNA transduction was quantitated by qPCR and normalized to GAPDH. Data shown represent 4 donors used in (C).

Since endogenous, IFN-induced, MX2 is able to interact with NPC proteins, we next investigated the role played by NUP214 and TNPO1 in endogenous MX2 function during the inhibition of HIV-1 by IFNα. An U87-MG CD4^+^ CXCR4^+^ derived cell line was engineered where the *MX2* alleles were inactivated using CRISPR-Cas9 genome editing, and the effect of NUP214 and/or TNPO1 depletion by siRNA treatment was analyzed. As a control, we used siRNAs targeting MX2, as well as a CRISPR-Cas9 control cell line (CTRL CRISPR). As expected, IFNα treatment of CTRL CRISPR cells resulted in a severe reduction in the percentage of infected cells (Fig 5A). SiRNA-mediated silencing of MX2 reduced the suppression of virus infection caused by IFNα by ~50% when compared with the CTRL siRNA. Importantly, knock-down of NUP214, TNPO1 or both proteins together significantly reduced the effect of IFNα on viral infectivity to levels similar to that seen in cells depleted of MX2 alone.

In the MX2 CRISPR cell line, the reduction of viral infection observed after IFNα treatment (and transfection with CTRL siRNA) was substantially reduced when compared to the CTRL CRISPR cells (4.3- versus 12.4-fold reduction, Fig 5A and 5B; note the absence of MX2 in the IFN-treated cells in Fig 5B according to immunoblot analysis). Critically, no further reduction of infectivity was observed in the MX2 CRISPR cells when MX2, NUP214, TNPO1 or NUP214/TNPO1 together were depleted by siRNA treatment. The only noticeable difference observed in these cells was a modest increase in infectivity in cells depleted of NUP214 and not treated with IFNα, which consequently registered as a slightly greater IFNα effect upon NUP214 depletion. Taken together, these results reconfirm that endogenous MX2 contributes to the inhibition of HIV-1 by IFNα, and demonstrate that the contributions of NUP214 and TNPO1 to the anti-viral effect of IFNα are dependent on MX2.

In an attempt to provide additional evidence for the role(s) of NUP214 and TNPO1 in MX2 in anti-viral activity using a more physiologically relevant model, we tested the effect of depleting these two proteins, as well as MX2, in primary CD4^+^ T cells from 4 donors by shRNA-mediated silencing. Using NL4.3/Nef-IRES-Renilla as the challenge virus, depletion of MX2 yielded clear relief from the effect of IFNα in 3 of 4 donors (Fig 5C). Upon targeting of TNPO1, all donors displayed a diminution in the effects of IFNα, whereas only 2 donors showed an effect following NUP214 knock-down. However, analysis of cellular mRNA levels revealed that the shRNA-mediated suppression of NUP214 transcripts was relatively inefficient, likely explaining its evidently weaker phenotypic effect in these primary cell cultures (Fig 5D). These data are in concordance with the data obtained in cell lines, and confirm the contributions of MX2, NUP214 and TNPO1 to the IFNα response against HIV-1 infection.

### Nuclear envelope targeting of MX2 requires both NUP214 and TNPO1

**Fig 6:**
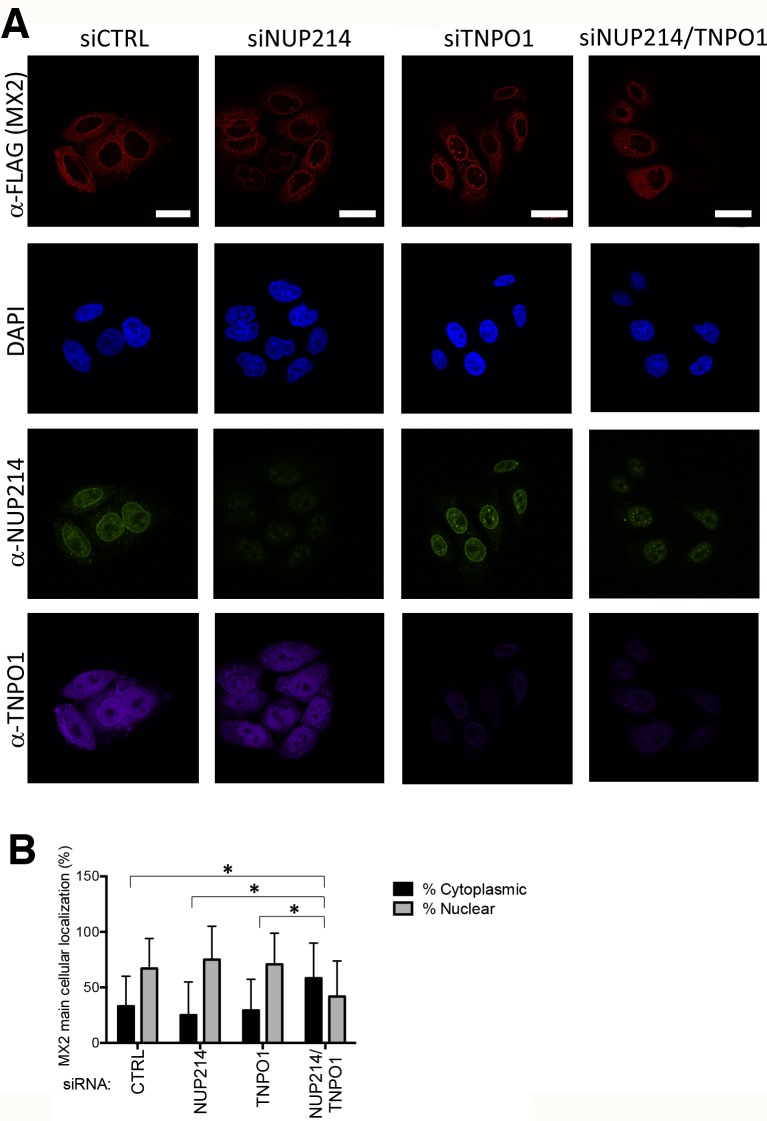
Depletion of NUP214 and TNPO1 disrupts nuclear envelope accumulation of MX2. **(A)** HeLa cells stably expressing MX2 bearing a C-terminal FLAG tag were transfected with siRNA targeting NUP214 and/or TNPO1 or a non-targeting siRNA (CTRL) as described in Fig 3, and then seeded onto glass coverslips. Localization of MX2, and endogenous NUP214 and TNPO1 was visualized by fluorescence confocal microscopy, using anti-FLAG, anti-NUP214, and anti-TNPO1 antibodies respectively. DAPI was used to stain the nuclei. Scale bar represents 20 μm. **(B)** Predominant cellular localization of MX2 was determined visually (blinded) using a 60x objective and an average of 100 cells, randomly selected (mean ± SD; *p-value < 0.05; paired t-test).

Human MX2 localizes at the NE [17, 39]; we therefore hypothesized that the molecular interactions described herein may facilitate tethering of MX2 to the cytoplasmic face of NPCs. Confocal microscopy was performed on HeLa cells stably expressing C-terminally FLAG-tagged MX2 and treated with control non-targeting siRNA or siRNAs specific for NUP214 and/or TNPO1 (Fig 6; panel B depicts visual scoring of MX2 localization for ~100 cells per sample). In cells treated with control siRNA, MX2 exhibited staining at the NE as well as diffuse staining throughout the cytoplasm [2, 17, 19]. NUP214 was also localized to the NE as in previous reports [26], and TNPO1 exhibited both nuclear and cytoplasmic staining as has also been described [40]. Treatment of cells with siRNA targeting either NUP214 or TNPO1 led to their respective depletion, but with no apparent effect on MX2 localization. However, in cultures doubly depleted for NUP214 and TNPO1, MX2 was significantly re-distributed to the cytoplasm with less pronounced NE accumulation. These findings infer that NUP214 and TNPO1 function in the NE targeting and localization of MX2; nevertheless, given that the single gene knock-downs yielded no significant alterations in MX2 localization, we speculate that the roles of each protein in NE targeting can be provided by redundantly acting host proteins.

## Discussion

**Fig 7:**
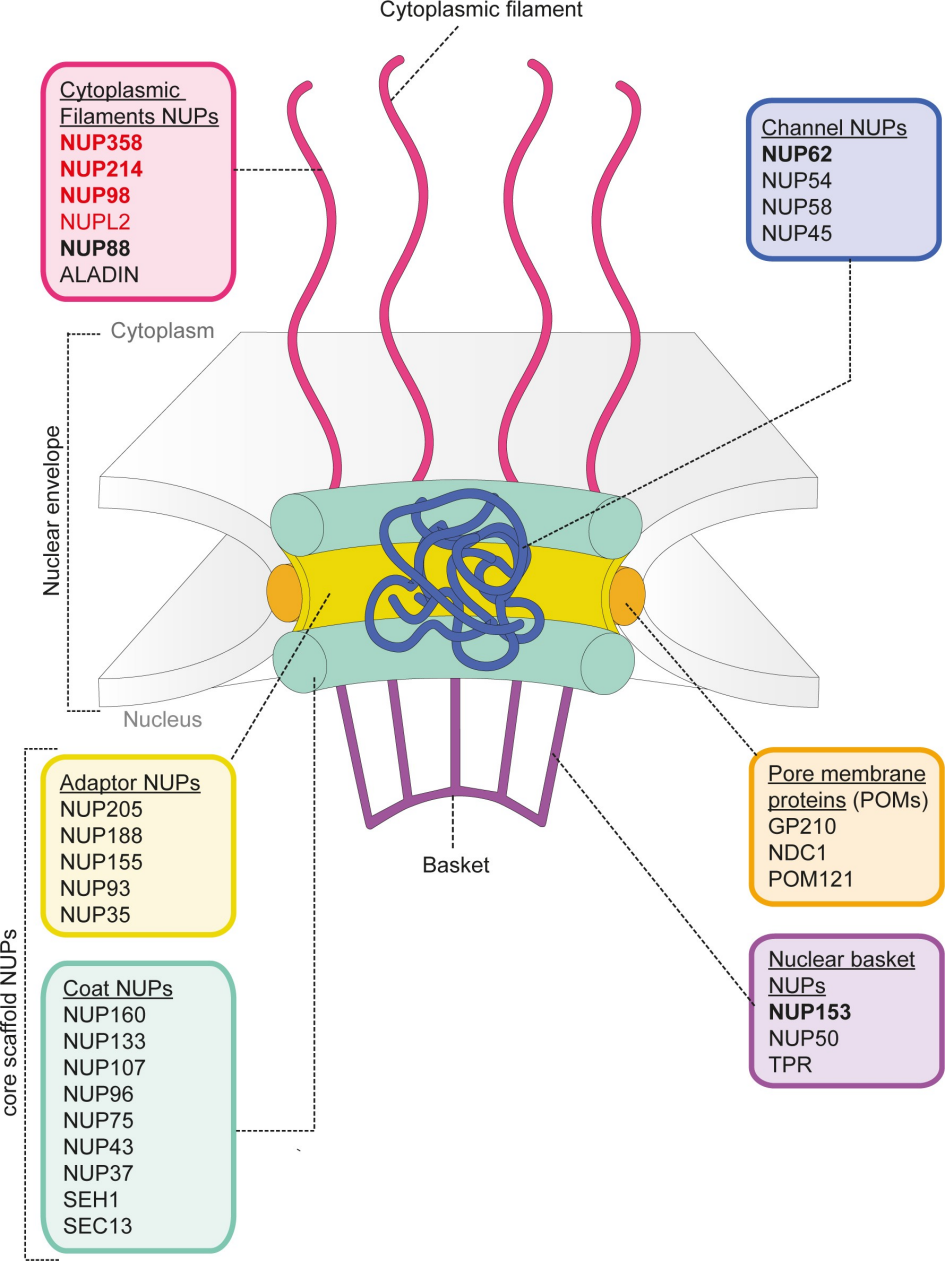
Nucleoporins interacting with MX2 are located at the cytoplasmic face of the nuclear pore. Schematic diagram indicating the structure and organization of the nuclear pore complex as described in Hoelz *et al* [22]. Nucleoporins interacting with MX2 (identified by Y-2-H screening) are shown in red. Nucleoporins required for MX2 activity (defined as those for which specific depletion elicited >50% reduction in MX2 dependent inhibition of HIV-1, in either HeLa or U87-MG cells) are shown in bold. Nucleoporins that interact with MX2 and are required for MX2 inhibition are shown in red and bold.

In seeking to define host co-factors required for MX2 inhibition of HIV-1, we started by performing an unbiased Y-2-H screen using the functionally essential N-terminal domain as bait. Remarkably, most of the candidate interactors with wild-type N-MX2 were members of a specific subfamily of nucleoporins, the cytoplasmic filament FG-nucleoporins (NUP358, NUP214, NUP98) or FG-repeat containing nucleoporin-like proteins also associated with the cytoplasmic face of the NE (NUPL2, hRIP) (Fig 1A; Fig 7). None of these candidates scored as interacting with the inactive mutant R11-13A thereby attesting to their likely biological relevance, and all interactions with wild-type MX2, with the exception of that with NUP358, were validated by co-immunoprecipitation (Fig 1B). Though we could not confirm the MX2—NUP358 interaction, data obtained through immunoprecipitation of endogenous nucleoporins with IFN-induced MX2 suggested that at least one other nucleoporin in addition to NUP214 on the cytoplasmic face of the NPC (which includes NUP358) may interact with MX2 (Fig 4A).

MX2 has previously been proposed to suppress HIV-1 infection by inhibiting the nuclear import of viral replication complexes, a view supported by the NE localization of the active 78 kDa isoform of MX2 [1, 2, 41]. Here, we have used RNA silencing to demonstrate that certain members of the family of cytoplasmic filament FG-nucleoporins are required for full-anti-viral activity of MX2 (Fig 2, Fig 3), including some that we have shown to interact with the MX2 N-terminal domain. In U87-MG cells, significant functional redundancy appeared to exist, although depletion of certain nucleoporins, particularly NUP214, as well as the transport receptor TNPO1, elicited a significant reduction in the potency of MX2-mediated inhibition (S1 Fig, Fig 2). Importantly, we further confirmed that NUP214 and TNPO1 are key determinants of the HIV-1 inhibitory function of endogenous MX2 during the IFNα response: depletion of NUP214 and/or TNPO1 significantly curtailed the anti-viral effect of IFNα, but only in cells expressing MX2 (Fig 5A and 5B). In addition, we also used RNA silencing to show, for the first time, that MX2 contributes to IFN-mediated suppression of HIV-1 in primary CD4^+^ T cells (Fig 5C). Consistent with our findings in cell line systems, silencing of TNPO1 or NUP214 (albeit to a lesser extent, and possibly due to less efficient silencing) also attenuated the effects of IFNα.

A considerable body of prior evidence has shown that HIV-1 requires the presence of specific nucleoporins to facilitate trafficking of viral replication complexes through the NPC (in studies where MX2 was not present) [24, 25, 42]. However, our experiments in U87-MG cells indicated that there is no specific requirement for particular nucleoporins for HIV-1 infection, implying effective functional redundancy between multiple nuclear import factors/pathways in MX2’s absence. In contrast, HIV-1 infection in HeLa cells exhibited a marked requirement for specific nucleoporins, particularly NUP358 and NUP153 and the transport receptor TNPO3, in agreement with previous observations (Fig 3) [24, 25, 42]. Perhaps mirroring such selectivity, a stronger dependence on certain nucleoporins and transport receptors for MX2-mediated inhibition was also observed in HeLa cells, with depletion of NUP214, NUP88, NUP358, NUP153 or TNPO3 eliciting ~4-fold reductions in MX2-mediated inhibition, and depletion of TNPO1, NUP62 and NUP98 eliciting ~3-fold reductions in MX2’s effect (Fig 3). However, the inherent dependence of HIV-1 infection upon NUP358, NUP153 and TNPO3 in HeLa cells confounds this analysis since their depletion results in less efficient infection regardless of MX2 expression. Taken together, observations in these cell lines suggest that multiple nucleoporins can contribute to MX2-mediated inhibition of HIV-1, but that significant functional redundancy exists and the requirements for specific nucleoporins vary between cell lines. Nevertheless, NUP214 and TNPO1 were consistently important for full MX2 anti-HIV-1 function, even in CD4^+^ T cells, and their depletion did not significantly impair infection in the absence of MX2: we therefore conclude that these proteins are particularly influential in promoting the anti-viral activity of MX2.

The pathway(s) through which nuclear import of HIV-1 proceeds remains unclear, as do the molecular details of inhibition by MX2. There is evidence to suggest that HIV-1 CA is a key specificity determinant in both processes [43]. Certain engineered mutations in CA permit escape from MX2-mediated restriction, including at positions P90 and G89 (the cyclophilin A (CYPA) binding loop), N74 and N57 (the CPSF6 binding site), and T210, N57 and G208 [1, 2, 18], However, current *in vitro* evidence indicates that such mutant CA proteins retain the ability to interact with MX2 [5, 7]. CYPA is required for MX2 inhibition in some cell lines [3] but not others, including CD4^+^ MT4 cells where CA mutant viruses that have lost the ability to interact with CYPA remain MX2 resistant [18]. Many of the same CA mutations that enable escape from MX2 have also been shown to influence HIV-1’s requirement for certain nucleoporins and transport factors during nuclear import. For instance, NUP358 can interact with CA directly through its cyclophilin domain, and mutations in CA at positions P90 and G89 reduce the dependence on both NUP358 and NUP153 for nuclear import in HeLa cells [24, 33]. The dependence of nuclear import on TNPO3 also appears to map to CA [44], with mutations at positions N74 and N57 within the CPSF6 binding site promoting use of a TNPO3 independent pathway [24]. However, the mechanism(s) through which these preferences exert their effects remains obscure.

Thus far, NUP214 has been primarily studied in the context of CRM-1 dependent nuclear export [26, 45], including that of HIV-1 viral RNAs [28]. However, NUP214 is required for docking of adenovirus (Ad) particles at the NPC and subsequent disassembly and nuclear import of the viral DNA [46]. Furthermore, through interaction assays with purified NEs from rat liver nuclei, and crosslinking studies with recombinant protein, the Ad2 capsid was shown to interact with the FG-repeat containing C-terminal domain of NUP214 [46] (also a site of MX2 interaction, Fig 1B). TNPO1 is a member of the karyopherin-β family, and while few previous studies have addressed its function in the HIV-1 life cycle, its role in nuclear translocation is believed to be restricted to import (reviewed in [47]). Many cargoes imported by TNPO1 possess a proline-tyrosine nuclear localization signal (PY-NLS) motif, a weak consensus sequence generally located within a basic and structurally disordered domain [48], with reported cargoes including CPSF6 and HIV-1 Rev [47]. Though the N-terminal domain of MX2 contains part of the consensus motif (R/H/X-X_2-5_-PY), no clear role for it in HIV-1 suppression is suggested since the inactive RRR11-13 mutant is still capable of binding to TNPO1 (albeit at reduced levels, Fig 4B, S2 Table), and mutation of residues within the consensus region do not interfere with anti-HIV-1 activity [19]. TNPO1, like other members of the karyopherin-β family, interacts with FG-repeat regions of nucleoporins, facilitating recruitment of these transport receptors and their associated cargoes to the NPC [47]. Earlier biochemical studies have also shown that recombinant TNPO1 can interact with NUP358, NUP214, NUP153, NUP98, and NUP62 from Xenopus egg extracts [49], but our studies failed to detect an interaction between TNPO1 and the C-terminal region of human NUP214 (consistent with a previous study [45]), despite the fact that the majority of NUP214’s FG-repeats are present within this region of the protein (Fig 4).

Taken together, this work invokes a model where NUP214 and TNPO1 are each important for the anti-viral activity of MX2, but that their contributions are both subject to degrees of cell type variation and redundancy, particularly in the case of NUP214 where additional FG-nucleoporins may serve similar role(s) in some cell types. Nevertheless, in light of their known functions and sites of localization (Fig 7), we propose a model in which interaction with TNPO1 helps recruit MX2 to the NPC, where MX2 can subsequently accumulate through interactions with NUP214 and other nucleoporins. The notion of redundant functionality among FG-nucleoporins for MX2 binding and localization is reminiscent of nucleoporin interactions with nuclear transport receptors [31, 50], and is illustrated by our Y-2-H and co-immunoprecipitation data (Fig 1), the sustained NE accumulation of the RRR11-13A mutant [19], as well as by the incomplete inhibition of MX2 function following efficient RNA silencing interventions (Fig 2, Fig 3).

Once localized at the NPC, MX2 is presumably then positioned to inhibit the nuclear import of HIV-1 nucleoprotein replication complexes [1, 2]. Current data suggest that the N-terminal domain of MX2 is again pivotal for activity, most likely via interactions with the viral CA protein [5, 17, 18, 20], thus adding a further interacting partner to the list of MX2 ligands. That said, it should also be acknowledged that a significant proportion of MX2 is cytosolic rather than NE localized (Fig 6) [17], and that interactions with viral replication complexes could initiate away from the NPC. One set of critical questions that is raised is: what are the specific roles(s) of the interactions between MX2, nucleoporins and CA, and how do they relate to one another in the context of viral inhibition? While nucleoporin binding to CA is thought to be important for HIV-1 translocation through the NPC, as well as ordered capsid disassembly (uncoating), MX2 interferes with these processes. Though this could be accomplished by MX2 competitively impeding CA-nucleoporin interactions, we favor the idea that MX2 inhibits HIV-1 infection through a specific process that targets viral replication complexes, a view that is supported by the noted ability of CA mutants to evade MX2. Another related question is: how does the N-terminal domain of MX2 accommodate a series of potentially sequential and competing interactions? A possible contributing solution is provided by MX2 oligomerization [5, 13], which may afford an MX2 oligomer the capacity to contact simultaneously different ligands, for instance, allowing CA binding while retaining NPC tethering. In sum, there is much to be resolved concerning the nature, orchestration and spatiotemporal ordering of the key molecular interactions and mechanistic processes that underpin MX2-mediated viral restriction at the NE.

### Note added during revision

During the review of this work, Kane *et al*., reported the use of RNA silencing to show that several nucleoporins impact HIV-1 infection as well as MX2 anti-viral function [51]. Our results are in good general agreement with their findings that depletion of NUP214 or TNPO1 impair MX2 anti-viral function in HeLa cells, and also with the apparently variable nucleoporin requirements for HIV-1 infection and MX2 activity among different cell lines.

## Materials and methods

### Cell culture and plasmid constructs

Human 293T cells, HeLa cells and parental U87-MG cells were obtained from the American Tissue Culture Collection (ATCC). The generation of the U87-MG CD4^+^ CXCR4^+^ cell line has been described [1]. All cell lines were cultured in Dulbecco’s modified Eagle’s medium supplemented with fetal bovine serum (10%), L-glutamine and penicillin-streptomycin. Human primary CD4^+^ T cells were obtained through the Infectious Diseases BioBank at King’s College London (ethics reference MM2-220518) and were isolated from peripheral blood mononuclear cells (PBMCs) of anonymous healthy volunteer donors with written consent under overall permission from the Southampton and South West Hampshire Research Ethics Committee (B) (approval REC09/H0504/39+5). CD4^+^ T cells were purified by density gradient centrifugation using LymphoPrep (Axis-Shield) and isolated by negative selection using the CD4^+^ T Cell Isolation Kit (Miltenyl Biotec) following manufacturer’s instructions. Activation of the cells was achieved using Dynabeads Human T-Activator CD3/CD28 (ThermoFisher) and 50 U/ml recombinant IL-2 (rIL-2) (Roche) for 48 h in medium consisting of RPMI 1640-GlutaMAX containing 10% heat-inactivated autologous serum, 100 U/ml penicillin and 100 U/ml streptomycin. After activation, cells were maintained in medium containing 30 U/ml of rIL-2. IFNα-2b (Merck, Sharpe & Dohme Corp.) was added to cultures for 24 h prior to infection, as indicated.

Construction of pCAGGS based expression constructs encoding FLAG- or HA-tagged GFP, MX1 or MX2 has been described previously [13, 17]. pCAGGS R11-13A MX2-HA was generated by replacing the cDNA encoding the first 25 amino acids from MX2-HA with the mutated version from R11-13 MX2-FLAG (described previously [19]) using NotI and EcoRI. To generate FLAG-NUP214 CTD (~40 kDa C-terminal fragment), the cDNA encoding the C-terminal amino acids (positions 1681–2080) of NUP214 (IMAGE clone BC105998) were PCR amplified with an N-terminal FLAG tag and cloned into pCAGGs. pCAGGs FLAG-KLHL6, PNRC1-FLAG, FLAG-NUPL2 and RUNX3-FLAG were generated by PCR amplification of full-length cDNAs from IMAGE clones BC032348, BC018112, BC107583, and BC013362 respectively. pCAGGs FLAG-TNPO1 and HA-TNPO1 were generated from PCR amplification of the full-length cDNA from IMAGE clone BC040340. FLAG-NUP98 and T7-hRIP were gifts from Drs Maria Teresa Catanese and Chad Swanson, respectively. EasiLV based lentiviral expression vectors (pRRL.sin.cPPT.(7TetOCMV/CDS.IRES.rtTA3-2A-E2-Crimson)_antisense_.WPRE) encoding MX2-FLAG or *Photinus pyralis* luciferase-FLAG; lentiviral vectors conferring puromycin selection (LV-puromycinR) (pRRL.sin.cPPT.CMV/CDS-IRES-puromycinR.WPRE) constitutively expressing CD8-FLAG or MX2-FLAG; and the NL4-3/nef-IRES-Renilla reporter virus (which expresses *Renilla* luciferase from an internal ribosome entry site, IRES) have been described [1, 13].

### Yeast two-hybrid screen

A yeast two-hybrid screen to probe for interaction partners with the N-terminal domain of MX2 was performed by Hybrigenics Services (ULTImate Y2H, www.hybrigenics-services.com). Amino acids 1 to 91 of human MX2 (N-MX2) were used as bait to screen against a human leukocyte cDNA library. Predicted interactions with native N-MX2 were compared to those identified using mutant RRR11-13A N-MX2 as a bait domain. Interactions were awarded a Predicted Biological Score (PBS) to assess the reliability of each interaction, primarily based on the number of independent prey fragments identified for each interaction compared to the probability of random selection [21]. For interactions awarded ‘A’ there is ‘very high confidence’ in the interaction, for those awarded ‘C’ there is ‘good’ confidence in the interaction, and those awarded F have been experimentally demonstrated to be technical artifacts.

### Co-immunoprecipitation

For co-immunoprecipitation of ectopically expressed affinity-tagged proteins, 293T cells were seeded in 6-well plates and co-transfected with pCAGGs based plasmids encoding triple-HA tagged and FLAG- or T7-tagged constructs using polyethylenimine. After ~30 h, cells were lysed in either hypotonic lysis buffer (10 mM Tris-HCl pH 8.0, 10 mM KCl, 1 × protease inhibitor cocktail (Roche)) by Dounce homogenization in experiments with PNRC1, KLHL6 and hRIP; or isotonic lysis buffer (1 × phosphate buffered saline, 0.5% Triton-X100, 1 × protease inhibitor cocktail) and lysed by sonication (20 s) for all other experiments. Lysates were cleared by centrifugation at 1,500 × g for 10 min, and for hypotonic lysates, KCl and Triton X-100 were added at final concentrations of 100 mM and 0.3%, respectively. A sample from each whole cell lysate was saved in order to confirm protein expression levels. HA-tagged proteins were immunoprecipitated using anti-HA magnetic beads (Pierce) for 2 h at 4°C, or FLAG-tagged proteins were immunoprecipitated with anti-FLAG (M2) magnetic beads (Sigma) under the same conditions. Beads were washed a further 4 times in wash buffer for hypotonic lysates (10 mM Tris-HCl pH 8.0, 200 mM KCl, 0.3% Triton X-100) or isotonic lysis buffer for isotonic lysates before the addition of sample buffer (200 mM Tris-HCl pH 6.8, 5.2% SDS, 20% glycerol, 0.1% bromophenol blue, 5% β-mercaptoethanol). HA-, FLAG- and T7 tagged proteins were resolved on 10% acrylamide gels by SDS-PAGE and detected by immunoblotting using HRP-conjugated anti-HA (rat monoclonal 3F10, Sigma), anti-FLAG (mouse monoclonal M2, Sigma), or anti-T7 antibody (mouse monoclonal, Sigma). Images were visualized by chemiluminescence (ECL western blotting substrate, Pierce) on a LI-COR Odyssey Fc imaging system (LI-COR) and band intensities of input and precipitated proteins were quantified using ImageJ software.

For co-immunoprecipitation of endogenous FG-nucleoporins and IFN-induced MX2, U87-MG CD4^+^ CXCR4^+^ cells were transfected twice, 24 h apart, with siRNAs targeting NUP214 (Dharmacon siGENOME smartpool) or a non-targeting control siRNA (Dharmacon). After 72 h, cells were treated with IFNα (500 U/ml) for 24 h to induce MX2 expression, prior to harvesting. Cells without IFN were included as a negative control for MX2 induction. Harvested cells were lysed in isotonic lysis buffer, sonicated, and the lysate cleared as described previously. FG-nucleoporins were immunoprecipitated with mouse monoclonal antibody mab414 [38] conjugated to protein G magnetic beads (Invitrogen) for 2 h at 4°C. An anti-GFP mouse monoclonal antibody (Roche) was used as a negative control. Beads were washed with isotonic lysis buffer, before addition of sample buffer. Immunoprecipitated endogenous FG-nucleoporins NUP358, NUP214, NUP153 and NUP62 were detected by immunoblotting with mab414 after SDS-PAGE on 6% acrylamide gels, and MX2 was detected by immunoblotting with anti-MX2 goat polyclonal antibody (N17, Santa Cruz Biotechnology). Bound primary antibodies were detected with HRP-conjugated anti-mouse or anti-goat immunoglobulin secondary antibodies and visualized by chemiluminescence.

### HIV-1 vector infectivity assays

For experiments in U87-MG CD4^+^CXCR4^+^ cells, ectopic expression of MX2 was achieved by transduction of cells with doxycycline-inducible EasiLV lentiviral vectors encoding FLAG-tagged human MX2 (or a FLAG-tagged luciferase control construct) for 6 h. After a rest period of 48 h, transduced cells were transfected twice, 24 h apart, with a panel of siRNAs (all human siGENOME smartpools, Dharmacon) at 20 nM concentration using Lipofectamine RNAiMAX reagent (Invitrogen). After 16 h, transgene expression was induced by the addition of 0.5 μg/ml doxycycline for 72 h, prior to viral challenge. EasiLV transduction efficiency was typically above 90% and was assessed by measuring the percentage of cells expressing E2-crimson (co-expressed via an IRES) by flow cytometry (FACSCanto II; BD Biosciences). To assess HIV-1 infectivity, cells were challenged with HIV-1/GFP, a vesicular stomatitis virus G protein (VSV G)-pseudotyped 8.91 HIV-1 Gag-Pol based cytomegalovirus (CMV) immediate early-enhanced green fluorescent protein lentiviral vector as described previously [13]. Productive infection was enumerated by flow cytometry as the percentage of E2-crimson-positive cells expressing GFP at 48 h post infection. Preparation of EasiLV particles and challenge HIV-1/GFP vector stocks has been described [1, 17]. In experiments measuring the effect of MX2, NUP214 and/or TNPO1 depletion by siRNA during an IFN response, CTRL CRISPR or MX2 CRISPR U87-MG cells were doubly transfected 24 h apart with 10 nM siRNA in 24-well plates. 8 h later, cells were split into two populations and seeded in 96 well plates. One population was treated with IFNα while the other one was left untreated. 24 h later cells were challenged with HIV-1/GFP as described.

For experiments in HeLa cells, constitutive overexpression of MX2 was achieved by transduction of cells with lentiviral vector LV-PuromycinR expressing FLAG-tagged human MX2 (or a FLAG-tagged CD8 control construct) [1]. After 72 h, transduced cells were selected with 1 μg/ml puromycin for a further 72 h (100% cell mortality for non-transduced cells). Selected cells were treated twice with siRNA, and challenged with HIV-1/GFP, as described previously. In both cell lines, efficiency of siRNA-mediated silencing prior to challenge was assessed by immunoblotting. Cell pellets were lysed in sample buffer, resolved by SDS-PAGE, and detected using the following primary antibodies: mab414 (FG-nucleoporins NUP358, NUP214, NUP153, NUP62), anti-NUP98 (rat monoclonal 2H10, Abcam), anti-hRIP (goat polyclonal, Santa Cruz Biotechnology), anti-KLHL6 (rabbit monoclonal, Abcam), anti-TNPO1 (mouse monoclonal D45, Abcam), anti-α-tubulin (mouse monoclonal, Sigma), anti-Hsp90 (rabbit; Santa Cruz Biotechnology), anti-NUP188 (rabbit polyclonal, Abcam), anti-TNPO3 (mouse monoclonal, Abcam), anti-NUP88 (rabbit polyclonal, Abcam), anti-hRIP (rabbit polyclonal, Abcam) or anti-PNRC1 (rabbit polyclonal, antibodies-online.com). Bound primary antibodies were detected with HRP-conjugated anti-mouse, anti-rat, anti-rabbit or anti-goat immunoglobulin secondary antibodies and visualized by chemiluminescence.

### CRISPR-Cas9 genome editing of U87-MG cells

U87-MG cells were transduced with VSV-G-pseudotyped HIV-1 lentiviral vector (LV) bearing plentiCRISPRv2. Specific guide RNAs targeting MX2 or red fluorescent protein (RFP) (as the control) were cloned into BsmBI-linearized lentiviral vector pLentiCRISPRv2 using the oligonucleotides (forward/reverse) caccgAATTGACTTCTCCTCCGGTA / aaacTACCGGAGGAGAAGTCAATTc for MX2 and caccgCTCAGTTCCAGTACGGCTCCA / aaacTGGAGCCGTACTGGAACTGAGc for RFP. Transduced cells were selected with 1 μg/ml puromycin. Single-cell clones were obtained by limiting dilution and grown in a 96-well plate in the absence of puromycin and MX2 depletion was validated by immunoblotting.

### shRNA-mediated gene silencing

Modified versions of the HIV-1 based lentiviral vector pHRSIREN-S-SBP-∆LNGFR-W [52] where the selectable marker for antibody-free magnetic cell sorting (SBP-∆LNGFR) was replaced by GFP (CTRL and TNPO1) or E2-crimson (MX2 and NUP214) for cytometric analysis of transduction were used for gene silencing in primary CD4^+^ T cells. The shRNA targeting sequences for MX2, NUP214 and TNPO1 were AAGATGTTCTTTCTAATTG, GGTGAGAATCTTTGACTCC and GCAAAGATGTACTCGTAAG, respectively. Lentiviral vectors were obtained by cotransfection of 293T cells with VSV-G, p8.91 and the modified pHSIREN-S vector at a ratio of 0.5:1:1.5, respectively. Supernatants containing lentiviral particles were concentrated by ultracentrifugation and human primary CD4^+^ T cells were transduced by spin-infection at 2000 x g for 2 h at room temperature. 48 h after transduction, 2.5 x 10^4^ cells/well were seeded in a 96 well plate and treated or not with IFNα. 24 h later, cells were challenged with 30 ng p24^Gag^ of NL4.3/Nef-IRES-Renilla at 2000 x g for 2 h at room temperature. After 48 h, infection was assessed by measuring Renilla luciferase activity on a luminometer.

### Fluorescence microscopy

HeLa cells stably expressing MX2 bearing a C-terminal FLAG-tag were obtained by transduction with LV-PuromycinR [1] encoding MX2-FLAG and selection with 1 μg/ml puromycin for 72 h. Stable cells were then transfected twice, 24 h apart, with 20 nM siRNA in 24-well plates as described above. Transfected cells were seeded onto coverslips at ~50,000 cells per well in 12-well plates; 24 h later, cells were washed with 1x phosphate buffer saline and fixed in 4% paraformaldehyde (EM Sciences) for 15 min, permeabilized with 0.2% Triton X-100 for 15 min, and blocked/quenched in buffer NGB (50 mM NH_4_Cl, 2% goat serum, 2% bovine serum albumin) for 1 h. MX2-FLAG was detected using a mouse anti-FLAG monoclonal M2 (Sigma) and secondary donkey anti-mouse antibody conjugated to Alexa 594 (Invitrogen). Endogenous NUP214 was detected using rabbit anti-NUP214 polyclonal antibody (Abcam ab70497) and secondary donkey anti-rabbit antibody conjugated to Alexa 488 (Invitrogen). Endogenous TNPO1 was detected using rat anti-TNPO1 monoclonal L5G3 (antibodies-online.com) and secondary goat anti-rat antibody conjugated to Alexa 647 (Invitrogen). DAPI (4’,6-diamidino-2-phenylindole) staining was used to demarcate the nucleus (0.1 mg/ml for 5 min) Cells were visualized using a Nikon A1 point-scanning laser confocal microscope (Nikon Instruments). Blinded quantification of MX2 cellular localization was determined visually for 100 randomly selected cells.

## Supporting information

S1 Fig Screen for co-factors required for full anti-viral activity of MX2 in U87-MG cells. U87-MG CD4^+^ CXCR4^+^ cells were transduced with EasiLV vectors expressing FLAG-tagged MX2 or Luciferase (control). After 48 h, transduced cells were transfected twice, 24 h apart, with a panel of siRNAs at a concentration of 20 nM. For the first siRNA transfection, cells were treated with specific siRNAs, most of these targeting candidate interactors from the Y-2-H screen (Fig 1) including NUP358, NUP214, NUP98, hRIP, PNRC1, KLHL6 and a non-targeting siRNA was included as a control (CTRL). For the second siRNA transfection, cells were treated with a panel of specific siRNAs targeting a number of nucleoporins and transport receptors in addition to the Y-2-H candidates. Expression of MX2 or Luciferase was then induced by treatment of cells with doxycycline (0.5 μg/ml) for ~72 h prior to challenge with a HIV-1 based lentiviral vector expressing GFP (HIV-1/GFP). Transduction efficiency was assessed 48 h post challenge by flow-cytometry. Data are representative of two independent experiments.
(TIF)

S2 Fig (Accompanies Fig 2). Efficiency of siRNA-mediated depletion of endogenous proteins in U87-MG cells. U87-MG CD4^+^ CXCR4^+^ cells were transfected twice, 24 h apart, with 20 nM siRNA targeting NUP358, NUP214, NUP153, NUP62, NUP98, hRIP, KLHL6, NUPL2 and PNRC1. After 72 h, protein levels were analyzed by immunoblotting, with α-tubulin or HSP90 included as loading controls. No reduction in target protein abundance was observed after treatment with siRNA targeting NUPL2, and PNRC1 expression was not detectable by immunoblot.
(TIF)

S3 Fig (Accompanies Fig 3). Efficiency of siRNA-mediated depletion of endogenous proteins in HeLa cells. HeLa cells were transfected twice, 24 h apart, with 20 nM siRNA targeting NUP358, NUP214, NUP153, NUP62, NUP98, hRIP, KLHL6, PNRC1, NUP88, NUP188, TNPO1, TNPO3 and NUP214 together with TNPO1 (and CTRL siRNA). After 72 h, protein levels were analyzed by immunoblotting, with α-tubulin included as loading control.
(TIF)

S1 Table (Accompanies Fig 1A). Complete list and PBS of known genes identified in the yeast-two-hybrid screens.
(DOCX)

S2 Table (Accompanies Figs 1B and 4B). Quantification of co-immunoprecipitation of FLAG-tagged NUP214-CTD, NUP98, NUPL2, RUNX3, PNRC1, KLHL6, hRIP or TNPO1 with HA-tagged GFP, MX1, MX2 or RRR11-13A MX2. Values represent the ratio between protein input (IN) and the protein detected in the IP, normalized against wild-type MX2 interactions.
(DOCX)
