# Supplementary figures and images for: Multiple components of the nuclear pore complex interact with the amino-terminus of MX2 to facilitate HIV-1 restriction
Source: PLoS Pathog. 2018 Nov 29;14(11):e1007408. doi: 10.1371/journal.ppat.1007408 (PMC6264145; doi:10.1371/journal.ppat.1007408)

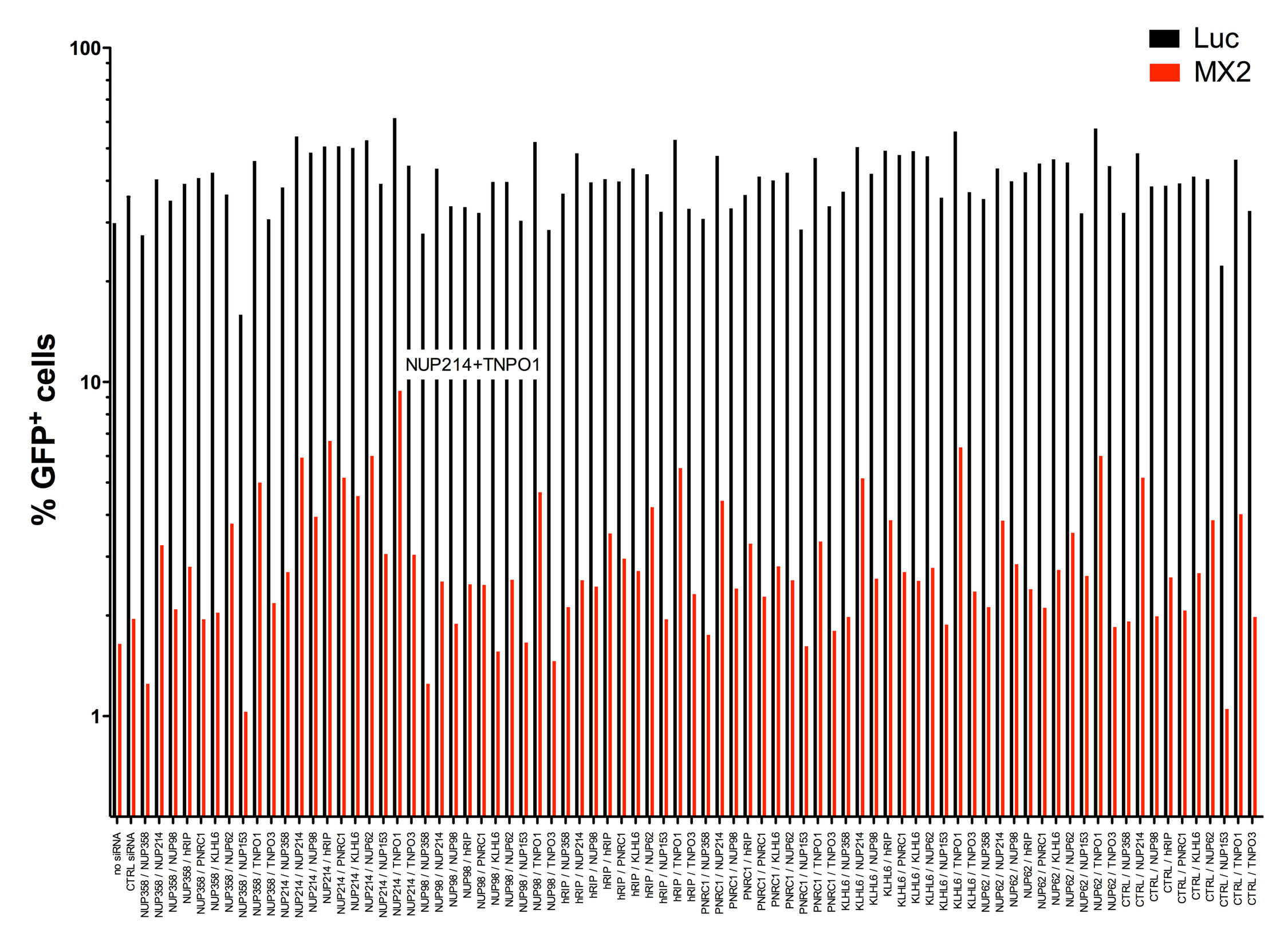

Supplement: S1 Fig — U87-MG CD4+ CXCR4+ cells were transduced with EasiLV vectors expressing FLAG-tagged MX2 or Luciferase (control). After 48 h, transduced cells were transfected twice, 24 h apart, with a panel of siRNAs at a concentration of 20 nM. For the first siRNA transfection, cells were treated with specific siRNAs, most of these targeting candidate interactors from the Y-2-H screen (Fig 1) including NUP358, NUP214, NUP98, hRIP, PNRC1, KLHL6 and a non-targeting siRNA was included as a control (CTRL). For the second siRNA transfection, cells were treated with a panel of specific siRNAs targeting a number of nucleoporins and transport receptors in addition to the Y-2-H candidates. Expression of MX2 or Luciferase was then induced by treatment of cells with doxycycline (0.5 μg/ml) for ~72 h prior to challenge with a HIV-1 based lentiviral vector expressing GFP (HIV-1/GFP). Transduction efficiency was assessed 48 h post challenge by flow-cytometry. Data are representative of two independent experiments. (TIF) [file ppat.1007408.s001.tif]

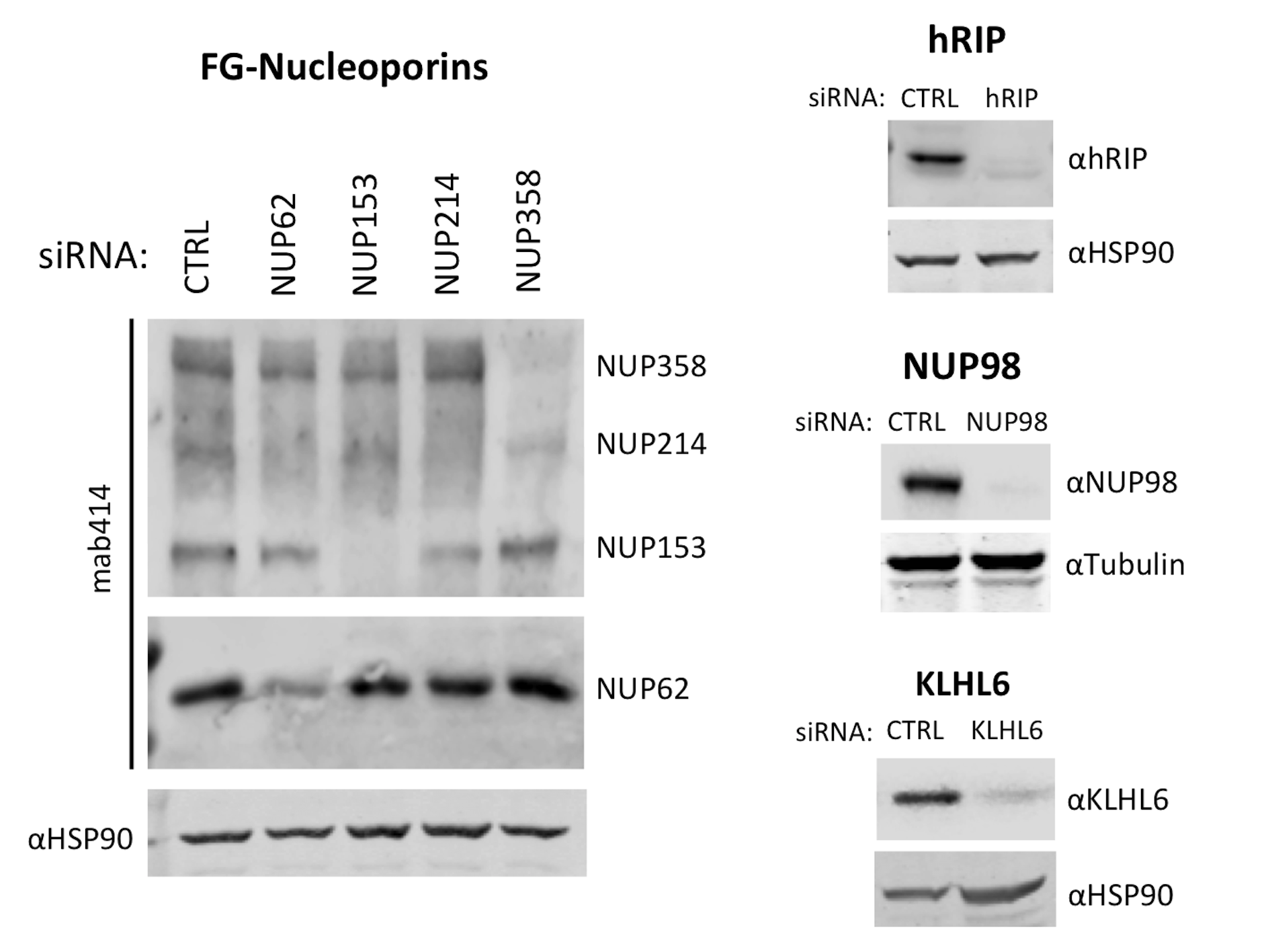

Supplement: S2 Fig — Efficiency of siRNA-mediated depletion of endogenous proteins in U87-MG cells. U87-MG CD4+ CXCR4+ cells were transfected twice, 24 h apart, with 20 nM siRNA targeting NUP358, NUP214, NUP153, NUP62, NUP98, hRIP, KLHL6, NUPL2 and PNRC1. After 72 h, protein levels were analyzed by immunoblotting, with α-tubulin or HSP90 included as loading controls. No reduction in target protein abundance was observed after treatment with siRNA targeting NUPL2, and PNRC1 expression was not detectable by immunoblot. (TIF) [file ppat.1007408.s002.tif]

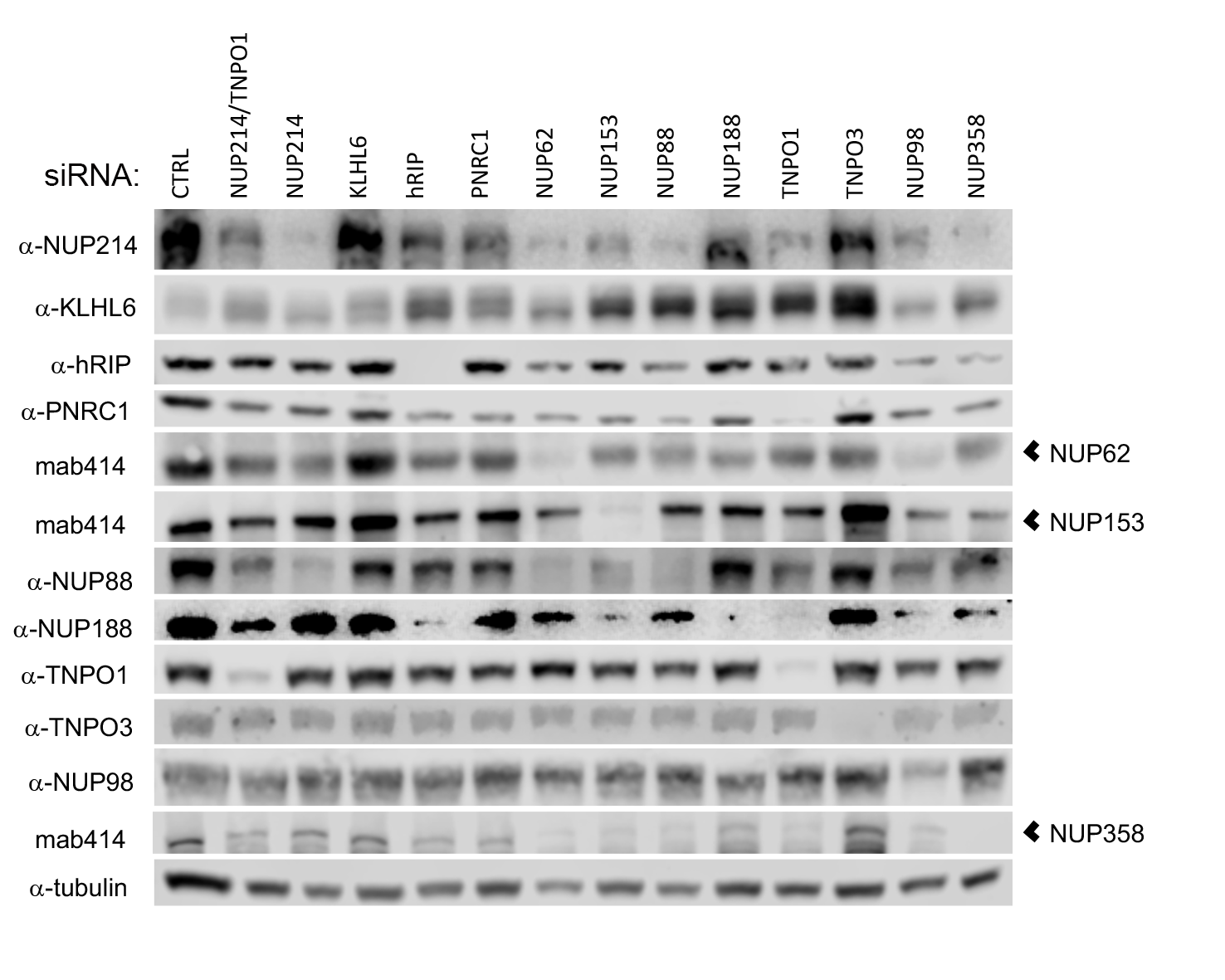

Supplement: S3 Fig — Efficiency of siRNA-mediated depletion of endogenous proteins in HeLa cells. HeLa cells were transfected twice, 24 h apart, with 20 nM siRNA targeting NUP358, NUP214, NUP153, NUP62, NUP98, hRIP, KLHL6, PNRC1, NUP88, NUP188, TNPO1, TNPO3 and NUP214 together with TNPO1 (and CTRL siRNA). After 72 h, protein levels were analyzed by immunoblotting, with α-tubulin included as loading control. (TIF) [file ppat.1007408.s003.tif]
