# Supplementary material for: Multiple components of the nuclear pore complex interact with the amino-terminus of MX2 to facilitate HIV-1 restriction
Source: PLoS Pathog. 2018 Nov 29;14(11):e1007408. doi: 10.1371/journal.ppat.1007408 (PMC6264145; doi:10.1371/journal.ppat.1007408)
Supplement: S1 Table — Complete list and PBS of known genes identified in the yeast-two-hybrid screens. (DOCX) [file ppat.1007408.s004.docx]

| **WT N_MX2_** | | | **RRR-11-13A N_MX2_** | | |
| --- | --- | --- | --- | --- | --- |
| **PBS** | **Gene name** | **Gene ID** | **PBS** | **Gene name** | **Gene ID** |
| A | AGFG1/hRIP | NM_004504.4 | B | RUNX3 | NM_001031680.2 |
| A | KLHL6 | NM_130446.2 | D | CTSZ | NM_001336.3 |
| A | NUP214 | NM_005085.3 | D | GMEB2 | NM_012384.3 |
| A | PNRC1 | NM_006813.2 | D | MBD1 | NM_015846.3 |
| A | RANBP2/ NUP358 | NM_006267.4 | D | POU2F2 | NM_001247994.1 |
| C | NUPL2 | NM_007342.2 | D | SEPT6 | NM_145799.3 |
| C | NUP98 | NM_005387.5 | D | STX11 | NM_003764.3 |
| D | ATRIP | NM_130384.2 | D | ZC3H15 | NM_001080412.2 |
| D | BIRC3 | NM_001165.4 | E | HIVEP2 | NM_006734.3 |
| D | HIATL1 | NM_032558.2 | E | KDM2A | NM_012308.2 |
| D | KANSL1 | NM_001193466.1 | E | PIAS1 | AF167160.1 |
| D | KIAA0355 | NM_014686.3 | E | TAF1 | NM_004606.4 |
| D | MAP2K1 | NM_002755.3 | E | ZBTB38 | NM_001080412.2 |
| D | MCM3AP | NM_003906.4 |  |  |  |
| D | PDE7A | NM_002603.3 |  |  |  |
| D | PNRC2 | NM_017761.3 |  |  |  |
| D | PPP1R16B | NM_015568.2 |  |  |  |
| D | PRKCAB | NM_182948.3 |  |  |  |
| D | RPS2 | NM_002952.3 |  |  |  |
