# Supplementary material for: Multiple components of the nuclear pore complex interact with the amino-terminus of MX2 to facilitate HIV-1 restriction
Source: PLoS Pathog. 2018 Nov 29;14(11):e1007408. doi: 10.1371/journal.ppat.1007408 (PMC6264145; doi:10.1371/journal.ppat.1007408)
Supplement: S2 Table — Quantification of co-immunoprecipitation of FLAG-tagged NUP214-CTD, NUP98, NUPL2, RUNX3, PNRC1, KLHL6, hRIP or TNPO1 with HA-tagged GFP, MX1, MX2 or RRR11-13A MX2. Values represent the ratio between protein input (IN) and the protein detected in the IP, normalized against wild-type MX2 interactions. (DOCX) [file ppat.1007408.s005.docx]

|  | GFP-HA* | MX1-HA* | MX2-HA* | A^11-13^-HA* |
| --- | --- | --- | --- | --- |
| FLAG-NUP214-CTD | 0 | 0 | 1 | 0.09 |
| FLAG-NUP98 | 0 | 0 | 1 | 0.31 |
| FLAG-NUPL2 | 0 | 0 | 1 | 1.16 |
| RUNX3-FLAG | 0.01 | 0 | 1 | 1.85 |
| PNRC1-FLAG | 0.02 | 0.13 | 1 | 0.08 |
| FLAG-KLHL6 | 0 | 0.03 | 1 | 1.27 |
| T7-hRIP | 0.03 | 0.04 | 1 | 0.61 |
| FLAG-TNPO1 | 0 | 0 | 1 | 0.43 |

*Values represented are the ratio between protein input (IN) and the protein detected in the IP, normalized against wild-type MX2 interactions.
